# Tracking outbreak populations of the pepper weevil *Anthonomus eugenii* (Coleoptera; Curculionidae) using complete mitochondrial genomes

**DOI:** 10.1371/journal.pone.0221182

**Published:** 2019-08-14

**Authors:** Bart T. L. H. van de Vossenberg, Tim Warbroek, Joseph Ingerson-Mahar, Cees Waalwijk, Lucas P. van der Gouw, Bernadette Eichinger, Antoon J. M. Loomans

**Affiliations:** 1 National Reference Centre of plant health, Dutch National Plant Protection Organization, Wageningen, Gelderland, the Netherlands; 2 Department of Entomology, Rutgers University, New Brunswick, New Jersey, United States of America; 3 Biointeractions and Plant health, Wageningen University & Research, Wageningen, Gelderland, the Netherlands; National Cheng Kung University, TAIWAN

## Abstract

The pepper weevil, *Anthonomus eugenii*, is a major pest on Capsicum species. Apart from natural spread, there is a risk of spread via international pepper trade. In the Netherlands, a pepper weevil outbreak occurred in 2012 and affected six greenhouses producing different sweet pepper varieties. The following year, a pepper weevil outbreak occurred in Italy. To trace the origin of the Dutch outbreak and to establish if the Dutch and Italian outbreaks were linked, we determined the mitogenomes of *A*. *eugenii* specimens collected at outbreak locations, and compared these with specimens from the native area, and other areas where the pest was introduced either by natural dispersal or via trade. The circular 17,257 bp *A*. *eugenii* mitogenome comprises thirteen mitochondrial genes typically found in insect species. Intra-species variation of these mitochondrial genes revealed four main mitochondrial lineages encompassing 41 haplotypes. The highest diversity was observed for specimens from its presumed native area (i.e. Mexico). The Dutch outbreak specimens represented three highly similar haplotypes, suggesting a single introduction of the pest. The major Dutch haplotype was also found in two specimens from New Jersey. As the Netherlands does not have pepper trade with New Jersey, it is likely that the specimens sampled in New Jersey and those sampled in the Netherlands originate from a shared source that was not included in this study. In addition, our analysis shows that the Italian and Dutch outbreaks were not linked. The mitochondrial genome is a useful tool to trace outbreak populations and the methodology presented in this paper could prove valuable for other invasive pest species, such as the African fruit moth *Thaumatotibia leucotreta* and emerald ash borer *Agrilus planipennis*.

## Introduction

The pepper weevil, *Anthonomus eugenii* Cano (Coleoptera: Curculionidae), is considered as one of the most important pests on cultivated peppers (*Capsicum* spp.), because of damage caused by direct feeding on the fruits [1]. Larvae feed on soft tissues inside the developing fruit, whereas adults feed on flowers and young fruits. Presence of the pest results in malformation, premature ripening and abscission of fruits [2], and losses of 33% to 100% have been reported [3, 4]. Cultivated peppers are the main host for *A*. *eugenii*, but the pest can also complete its lifecycle on several other solanaceous species, such as: *Solanum melongena* (egg-plant), *Solanum rostratum* (buffalobur nightshade) and *Solanum nigrum* (black nightshade) [5]. Because of its impact, *A*. *eugenii* is listed as quarantine pest in several European, South-American and African countries [6].

Based on the earliest reports of the pepper weevil and its hosts, it is generally believed that the species originates from Mexico or surrounding regions in Central America [7]. Today, *A*. *eugenii* is a major pest species on peppers in Mexico, the southern United States, central America, and the Caribbean [8]. Apart from its natural range, the pest has been found outdoors in Hawaii in 1933 [1], French Polynesia pre-1986 [9], Puerto Rico in 1982 [10], and the Dominican Republic in 2006 [11]. The pest was also encountered in greenhouses in southern Canada in 1992 [12] and again in 2009 and 2010 [13]. Similarly, the pest was found in greenhouses in the Netherlands (2012) [14] and Italy (2013) [15], where in the latter country the pest was also found outdoors. In these cases, the infestations are believed to be associated with the import of *Capsicum* and/or *Solanum* fruits, or plants for planting.

For the European Union the risk of infestation from the import of *Capsicum* and *Solanum* fruits was initially regarded as low (but with a high uncertainty), as import of host fruits from countries in which the pest is endemic occurs since at least 1988 and no outbreaks had taken place since [16]. In the Netherlands, *A*. *eugenii* was first intercepted on eggplant fruits from the Dominican Republic in 1999 [17], and since then the pest has been intercepted numerous times from Mexico and the Dominican Republic at import inspections. The first and only outbreak of *A*. *eugenii* in the Netherlands was in July 2012 when the pest was found in four greenhouses producing sweet pepper (*Capsicum annuum*) fruit in the province of Zuid-Holland [14]. In October and November of the same year, two additional greenhouses were found to be infested. All infested greenhouses were situated close to each other (2 km radius) and a surveillance program was initiated. Eradication measures were taken against *A*. *eugenii* which included the application of pesticides, and the destruction and secured removal of plants and growing medium in all infested greenhouses. The National Plant Protection Organization declared that the pest was successfully eradicated in the Netherlands in December 2013 [18]. In Italy, the pest was declared eradicated in 2018 [19].

The mitochondrial genome is frequently used in phylogenetic [20, 21] and population-level studies [22, 23] as it typically represents a single maternally inherited molecule with low levels of recombination while being present in high copy numbers [24, 25]. With the introduction of next generation sequencing technology, the number of annotated complete mitogenomes has increased tremendously [26]. For instance, higher level phylogenetic relationships in the weevils (Curculionoidea), were determined by generating and analyzing close to one hundred (near) complete mitochondrial genomes from a single Illumina MiSeq run [27]. For the weevil tribe Anthonomini, one complete mitogenome, namely that of *Bradybatus kellneri* (KX087247), and two partial mitogenomes, those of *Anthonomus pomorum* (JN163951) and *Anthonomus grandis* (MG253256) are present in the public domain. To place the *A*. *eugenii* intraspecies variation in the context of other *Anthonomus* species, the mitogenomes of specimens of *A*. *pomorum*, *Anthonomus rectirostris* and *Anthonomus rubi* were assembled and included in this study.

## Material and methods

### Insect specimens

Following the Dutch 2012 outbreak, 32 specimens of *A*. *eugenii* were obtained from the affected greenhouses. Over fifty production sites of sweet pepper (*C*. *annuum*) fruit in an area of 4 x 9 km around the infested facilities were included in a follow-up survey in which pheromone traps (male aggregation pheromone in combination with a yellow sticky trap) were placed in primary production locations. These traps were also placed in greenhouses producing tomatoes, egg-plants or ornamental Solanaceae within an area of 2 x 3 km around the infested sites.

In addition, specimens from regular import inspections performed by the Dutch National Plant Protection Organization (NPPO-NL), and from collections as well as and specimens obtained from external contacts were included to create a reference panel of 135 *A*. *eugenii* specimens. Three *A*. *piri*, six *A*. *pomorum*, three *A*. *rectirostris* and four *A*. *rubi* specimens, all native to the Netherlands, were included resulting in a total of 151 *Anthonomus* spp. specimens (Table 1 and S1 Table). Specimens were identified morphologically [28] and selected specimens were also identified molecularly through Sanger sequencing of the partial mitochondrial *cox1* gene using primers LCO1490/HCO2198 [29] according to the EPPO DNA barcoding standard for selected plant pests [30].

**Table 1:** Overview of *Anthonomus* specimens included in this study.

Species	Origin (country)	specimens included
*Anthonomus eugenii* Cano, 1894	Dominican Republic	46
	Italy	2
	Mexico	29
	The Netherlands	32
	United States	26
*Anthonomus piri* Kollar, 1837	The Netherlands	3
*Anthonomus pomorum* Linnaeus, 1758	The Netherlands	6
*Anthonomus rectirostris* Linnaeus, 1758	The Netherlands	3
*Anthonomus rubi* Herbst, 1795	The Netherlands	4

### DNA extraction and sequencing

Genomic DNA was extracted from whole or partial *Anthonomus* spp. specimens on a KingFisher Flex (ThermoFisher, MA, USA) using the QuickPick SML genomic DNA Kit (Bio-Nobile, Finland). Insect tissue was ground in lysis buffer with a micro-pestle prior to DNA extraction. DNA was eluted in 100 μL elution buffer, and aliquots were sent to GenomeScan (Leiden, the Netherlands) for NextGen sequencing with the NextSeq 500 V2 platform under ISO17025 accreditation (Dutch Accreditation Body, scope L518). After fragmentation of the genomic DNA with the Bioruptor Pico (Diagenode, Belgium), sequencing libraries were prepared with the NEBNext Ultra DNA Library Prep kit for Illumina following manufacturer’s instructions. Each sample was tagged to create an unique index-flowcell combination, and libraries were split over four sequencing lanes in one of six flowcells resulting in a median yield of 2,463 Mb per sample (S1 Table).

### Assembly and annotation of mitogenomes

NextSeq reads of the *Anthonomus* spp. specimens were individually assembled *de novo* in CLC Genomic Workbench v.11 (default settings) and assembly errors were corrected using a read mapping approach (length fraction: 0.8, similarity fraction: 0.9). Putative mitochondrial scaffolds were identified by querying the genomic scaffolds against the *Anoplophora glabripennis* mitogenome (NC_008221) using blastn (e-value cut-off < 1e-20). For each of the *Anthonomus* species included, a MAFFT v7.388 alignment [31] (algorithm: G-INS-i) was created from the putative mitochondrial scaffolds to generate a consensus sequence per species. For each *Anthonomus* species, a single specimen was selected as reference for the species and final circular mitogenomes were reconstructed in GRAbB [32] (assembler = SPAdes v3.6; k-mer settings = 31, 61, and 91; run setting = exonerate) using the consensus mitogenomes as bait. The circular reference mitogenomes were annotated using the online MITOS tool using the invertebrate mitochondrial code [33]. Mitogenomes of the reference specimens were submitted to NCBI under accession numbers MK654676 (*A*. *eugenii*), MK654677 (*A*. *pomorum*), MK654678 (*A*. *rectirostris*), and MK654679 (*A*.*rubi*). The circular conformation of the *A*. *eugenii* mitogenome was verified by PCR amplification of the entire mitogenome in eight fragments. Reaction mixes were based on Phusion High Fidelity DNA polymerase (NEB, MA, USA) reagents using the primers and thermocycler profiles are described in S2 Table.

### Phylogenetic analysis

For each of the specimens included, NextSeq reads were mapped to the annotated reference mitogenome sequence of the respective species in CLC genomic Workbench (length fraction: 0.8, similarity fraction: 0.9). Annotations were transferred from the reference sequences to the resulting specimen specific sequences. Mitochondrial coding sequences for specimens with ≥10x mean coverage and 100% coverage relative to the reference sequence were used for phylogenetic and haplotype analyses. Datasets of fourteen specimens did not meet these requirements and were excluded from the analysis. Gene sequences of *atp6*, *atp8*, *cytb*, *cox1*, *cox2*, *cox3*, *nad1*, *nad2*, *nad3*, *nad4*, *nad4L*, *nad5*, and *nad6* from the specimens included in the analysis were submitted in NCBI under accession numbers MK652895 to MK654675.

Extracted mitochondrial coding sequences representing in total 137 *A*.*eugenii*, *A*. *pomorum*, *A*. *rubi* and *A*. *rectirostris* specimens were used, together with those of the publicly available outgroup *Bradybatus kellneri* (KX087247), to create a MAFFT v7.388 alignment (algorithm: FFT-NS-1) for each of the thirteen mitochondrial protein coding genes. Alignments were concatenated and a Maximum Likelihood (ML) phylogeny was constructed using CLC Genomic Workbench (Neighbour Joining with Jukes Cantor substitution model, and 500 bootstrap replications). The ML tree was provided with scientific name and origin of the specimens, and for *A*. *eugenii* specimens, the (major) mitochondrial haplogroups as defined below.

### Haplotype analysis

A concatenated alignment of the thirteen mitochondrial genes of 127 *A*. *eugenii* specimens was used to create a Median Joining network (ε = 0) using Popart v1.7 [34]. Haplotypes separated by >50 mutations were used to identify main haplogroups (1 to 4), which were further differentiated in subgroups, indicated with Latin letters, when separated by >5 mutations (e.g. 1A, 1B, 1C). Individual haplotypes within a subgroup were numbered sequentially (e.g. 1A1, 1A2, 1A3). In this systematic approach, the haplotype closest to the other major haplogroup was define as haplotype “A1”.

## Results

### Sampling

Following the 2012 Dutch outbreak, 70 specimens were collected from the six outbreak locations, referred to as greenhouse 1 to 6. No *A*. *eugenii* specimens were found in the follow-up survey including greenhouses producing *C*. *annuum* fruits, nor in greenhouses growing tomatoes, eggplants and ornamental solanaceous species around the infested greenhouses.

### Assembly and annotation of mitogenomes

**Fig 1:**
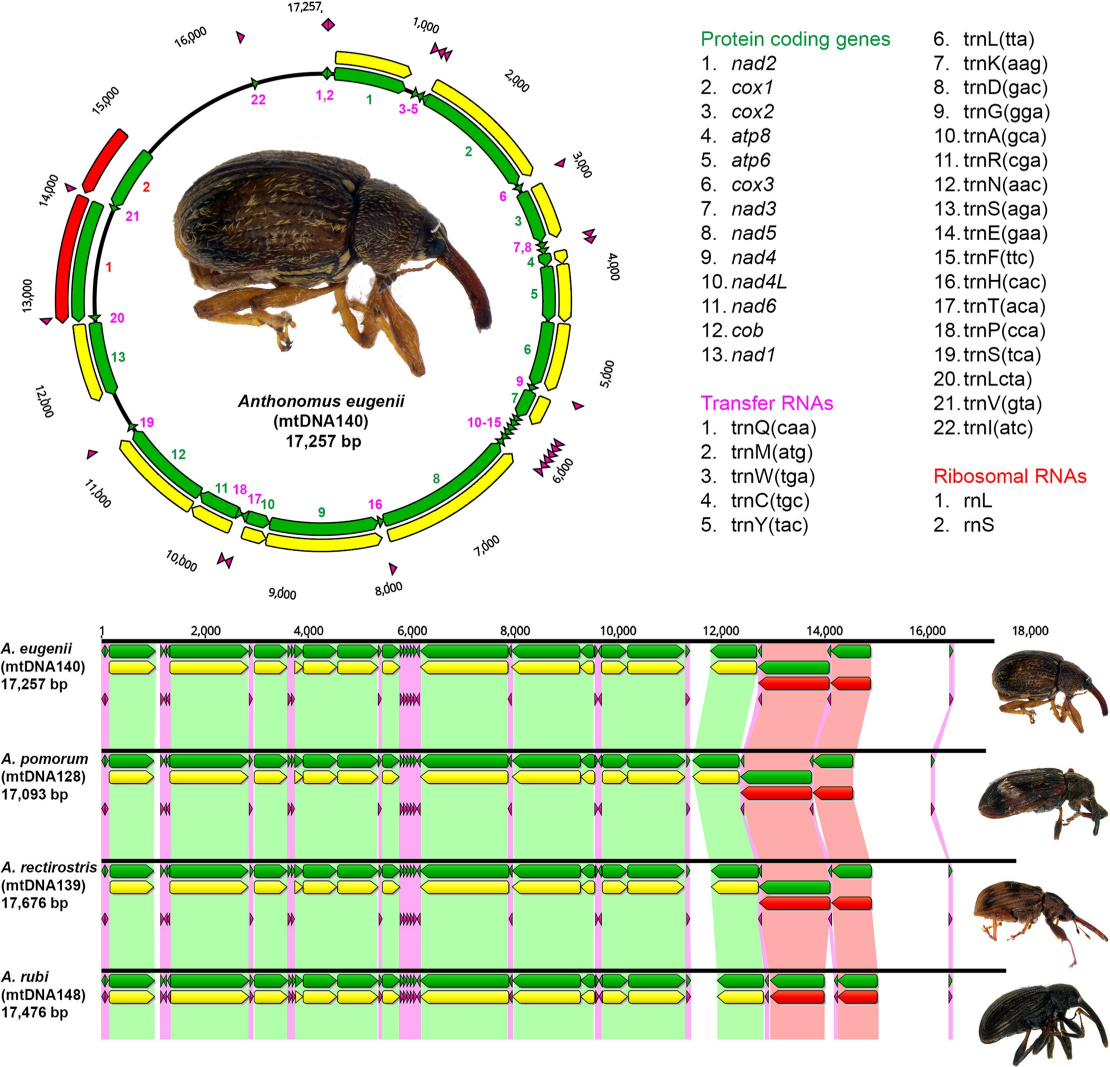
The *Anthonomus eugenii* mitogenome. **Top:** The *A*. *eugenii* mitogenome (17,257 bp) encoding thirteen protein coding genes (annotated in green, coding sequences are annotated in yellow), twenty-two transfer RNAs (purple), and two ribosomal RNAs (red). **Bottom:** comparison of the *A*. *eugenii*, *A*. *pomorum*, *A*. *rectirostris* and *A*. *rubi* mitogenomes (unaligned) shows the conserved organization of all mitochondrial genes. Orthologous elements are linked, using green links for protein coding genes, purple links for tRNAs, and red links for rRNAs.

NextGen sequence data was generated for all of the 151 *Anthonomus* specimens included in this study, and *de novo* assembly of this data resulted in 26,625 scaffolds on average. For *A*. *eugenii*, putative mitogenomic scaffolds were identified in 104 of the 127 specimens sequenced, which were used to create a chimeric mitochondrial consensus sequence. The chimeric sequence served as bait in the reference-guided GRAbB assembly for *A*. *eugenii* specimen mtDNA140, which was the first specimen found in the 2012 Dutch outbreak and serves as reference for the species. The GRAbB assembly for this specimen resulted in a 17,257 bp circular mitogenome with 413.9x mean coverage. The *A*. *eugenii* mitogenome encodes thirteen protein coding genes which are typically found on insect mitogenomes, i.e. genes encoding ATP synthase F0 subunits 6 and 8 respectively (*atp6*, *atp8*); cytochrome c oxidase subunits I, II, and III respectively (*cox1*, *cox2*, *cox3*); cytochrome b (*cob*), NADH dehydrogenase subunits 1–6 and 4L (*nad1*, *nad2*, *nad3*, *nad4*, *nad4L*, *nad5*, *nad6*). In addition, genes coding for the large and small ribosomal RNA subunits, and 22 transfer RNAs were identified on the *A*. *eugenii* mitogenome (Fig 1).

Similarly, chimeric mitochondrial consensus sequences were created for *A*. *pomorum*, *A*. *rectirostris* and *A*. *rubi*, which served as bait in a GRAbB assembly to create a single reference mitogenome for each of these species (Table 2). For *A*. *piri*, no complete mitogenome could be constructed on account of poor data obtained for the specimens included, and this species was excluded from further analyses. The complete mitogenomes of the four reference specimens show identical gene, rRNA and tRNA content, with conserved organization and orientation (Fig 1), and an overall GC-content ranging from 25.5% to 26.3% was observed.

**Table 2:** Reference *Anthonomus* spp. specimens.

Species	Specimen	Length (bp)	NCBI accession mitogenome	Mean coverage
*Anthonomus eugenii*	mtDNA140	17,257	MK654676	413.9
*Anthonomus pomorum*	mtDNA128	17,093	MK654677	86.7
*Anthonomus rectirostris*	mtDNA139	17,676	MK654678	203.2
*Anthonomus rubi*	mtDNA148	17,476	MK654679	134.8

The *A*. *pomorum*, *A*. *rectirostris* and *A*. *rubi* mitogenomes are characterized by 80–186 bp repeat sequences in the trnI—trnQ intergenic region, which do not share homology between the different species. For *A*. *eugenii*, no repetitive sequences were assembled in the trnI—trnQ intergenic region, but read-mapping and PCR based verification of the *A*. *eugenii* mitogenome indicate the presence of an unresolved repeat sequence in that region. Based on the primer sites designed with the 17,257 bp *A*. *eugenii* mitogenome, a 1,311 bp amplicon was expected for primer pair F6/R6, but surprisingly a ~3.5 kbp fragment was obtained. The amplicons lengths obtained for the other primer pairs corresponds with the *in silico* design (S1 Fig). In addition, read mapping of the *A*. *eugenii* reference specimen mtDNA140 to the reference mitogenome shows a sharp increase in the read coverage in the trnI-trnQ intergenic region on position 16,956 to 17,129 (2,176x mean coverage compared to an overall mean of 1,201x; S2 Fig). This suggests a repetitive sequence is present in the trnI–trnQ intergenic region which was not resolved in the GRAbB assembly.

### Phylogenetic reconstruction

**Fig 2:**
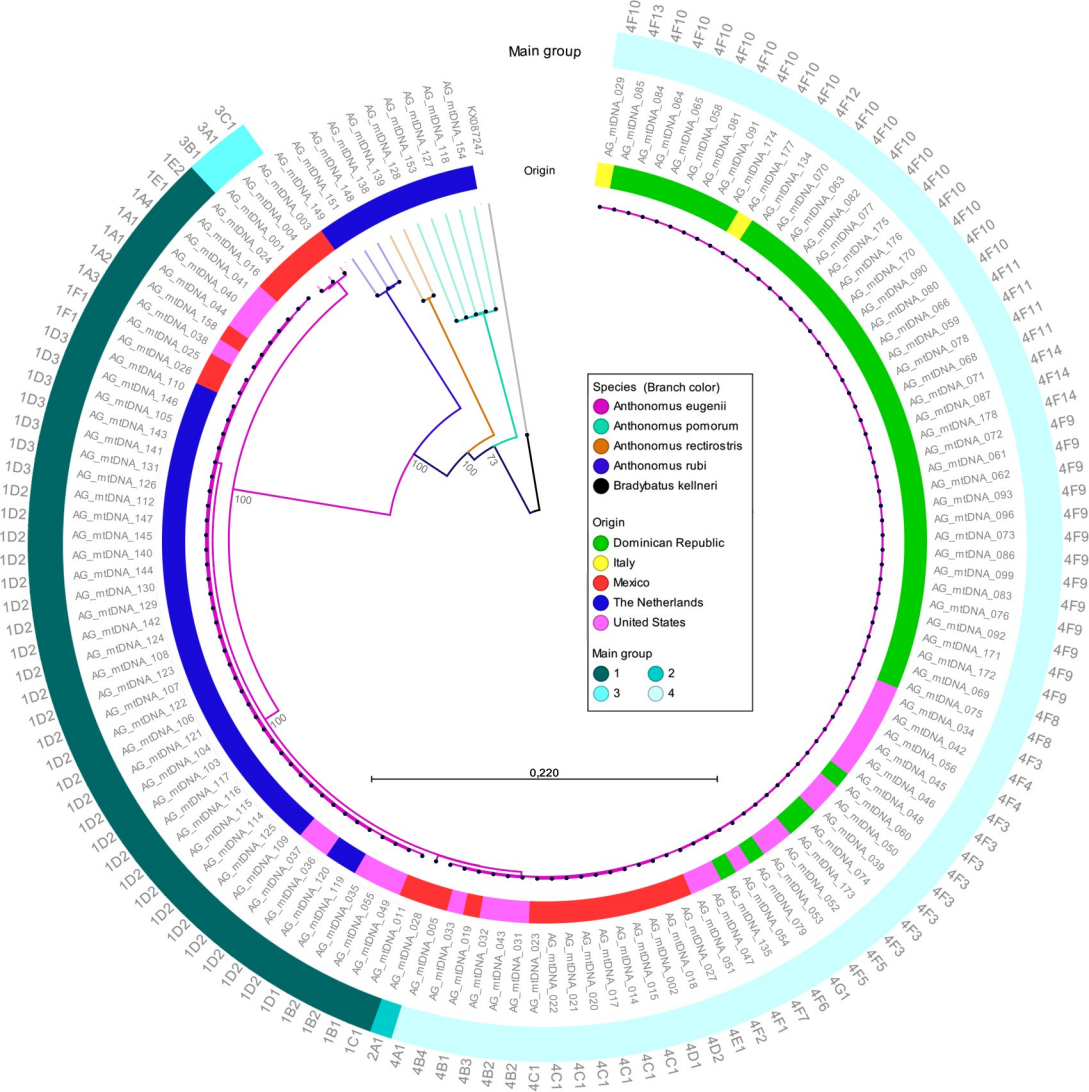
Phylogenetic reconstruction of *Anthonomus* specimens. Maximum Likelihood cladogram based on thirteen mitochondrial protein coding gene sequences of four *Anthonomus* species using *Bradybatus kellneri* as outgroup. Branches are colored according to species identity and bootstrap values are shown for the major nodes. The inner colored ring represents the country of origin, whereas the outer colored ring represents the main *A*. *eugenii* haplogroups. The specimen names are displayed as labels on the inner node layer and *A*. *eugenii* haplotypes are shown as labels on the outer node layer.

The thirteen protein coding mitochondrial genes were used to determine the phylogenetic relation between the 137 *A*.*eugenii*, *A*. *pomorum*, *A*. *rubi* and *A*. *rectirostris* specimens analyzed. The resulting concatenated MAFFT alignment consisted of 11,581 nt, including gaps. Gaps in the alignment were the result of gene length differences which were manually verified. Intra-species variation observed for the protein coding genes in *A*. *eugenii* consisted of single nucleotide polymorphisms and ranged up to 2,6%. Interspecies variation between *A*. *eugenii* and *A*. *pomorum*, *A*. *rubi*, or *A*. *rectirostris* ranged from 22.9 to 24.0%. Maximum Likelihood clustering of the mitochondrial protein coding sequences grouped specimens with the same morphological species identity with 73 to 100% bootstrap support. Clustering of *A*. *eugenii* specimens followed the main haplogroups as obtained in the haplotype network analysis (Fig 2).

### Haplotype network

**Fig 3:**
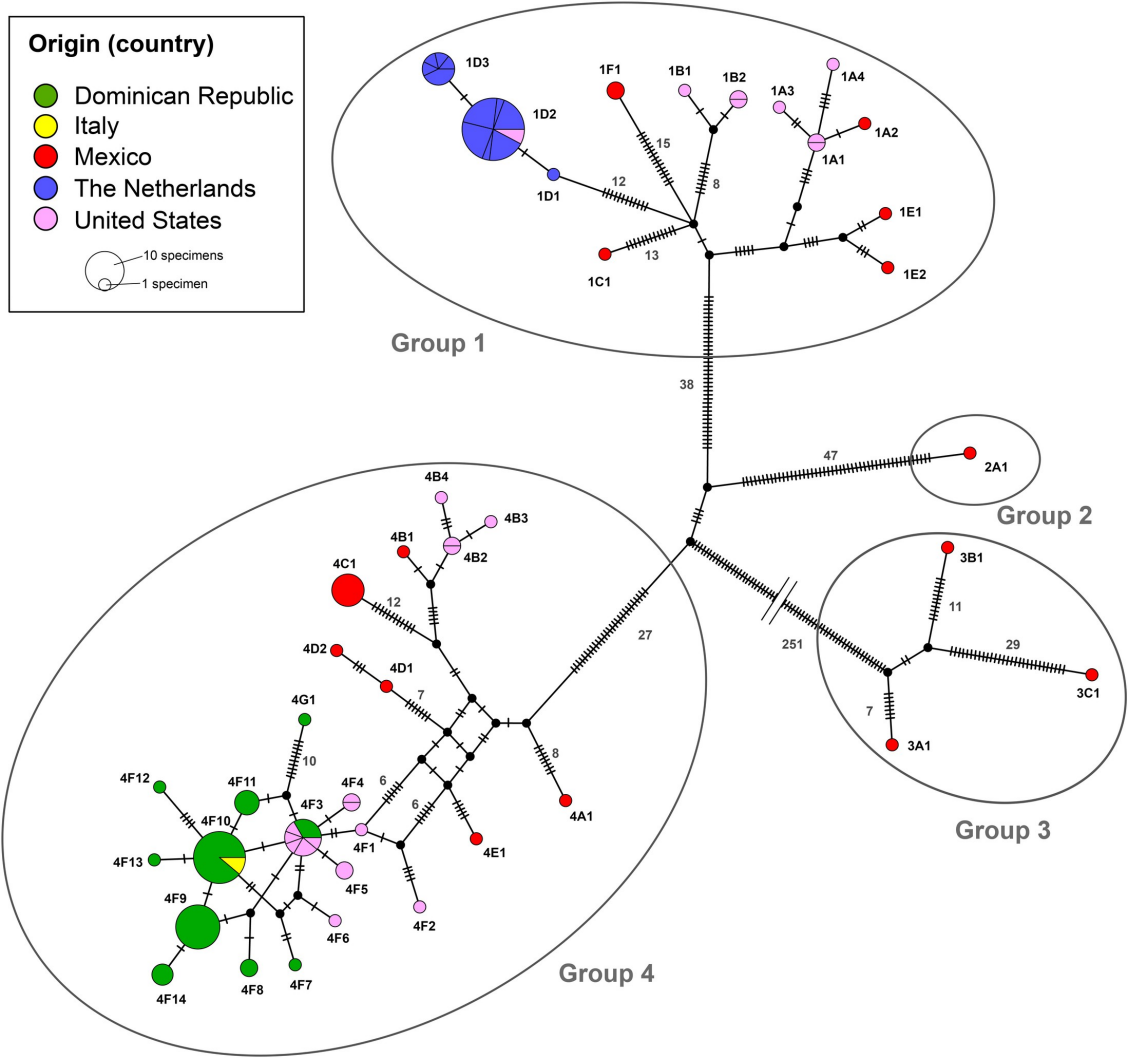
*Anthonomus eugenii* within-species diversity. Median Joining haplotype network representing 127 *A*. *eugenii* specimens based on 383 parsimony informative sites in the thirteen complete mitochondrial protein coding gene sequences. Nodes in the network are colored based on the origin of the samples, and black nodes represent hypothetical ancestors. Haplogroups are subdivided when they represent multiple localities of the same country. Marks on the branches indicate the number of mutations, where numbers are shown on branches with > 5 mutations. Main haplogroups 1 to 4 are separated by >50 mutations, while subgroups (e.g. A, B, C) are defined by >5 mutations.

Read mapping of NextSeq data of 135 *A*. *eugenii* specimens to the *A*. *eugenii* reference mitogenome resulted in complete mitochondrial sequences with >10x mean coverage for 127 specimens. A median joining haplotype network was created from the concatenated alignments of the thirteen mitochondrial protein coding genes. The resulting alignment comprised 10,914 nt and contained 383 parsimony informative sites. Haplotype analysis shows that the *A*. *eugenii* specimens cluster in four major groups that are separated by more than 50 mutations (Fig 3). A total of 41 unique haplotypes was obtained, of which fifteen consisted of more than one representative specimen (S3 Table).

The specimens from Mexico display the highest diversity and are represented by 15 haplotypes covering all four major haplogroups. Specimens collected in the province of Jalisco cluster in haplogroups 1 and 4, whereas specimens from Chihuahua are found in both haplogroups 3 and 4 (S3 Fig). Only single specimens from Tamaulipas and Aguascalientes were included in the study, which represent haplotypes in haplogroups 1 and 3, respectively. No locality information was available for other Mexican specimens which represented haplotypes in haplogroups 1, 2, and 4, and were obtained from regular import inspections. The high diversity displayed by the Mexican specimens is almost matched by specimens from New Jersey (USA) which were present in two major haplogroups and 14 different haplotypes. No link was identified between the sampling locality and the haplotype identity for samples from New Jersey and Georgia (S4 Fig) Specimens from the Dominican Republic were confined to subgroups 4F and 4G which represented 10 different haplotypes. The two specimens from the 2013 Italian outbreak were found in haplotype 4F10, and cluster with 18 specimens from the Dominican Republic.

All 32 specimens collected at the Dutch 2012 outbreak locations cluster in subgroup 1D, which consists of three haplotypes (i.e. 1D1, 1D2, 1D3). The majority of specimens (*n* = 24) belongs to haplotype 1D2, followed by haplotypes 1D3 (*n* = 7) and 1D1 (*n* = 1). Haplotype 1D1 differs a single nucleotide from haplotypes 1D2 and 1D3 on position 37 (A>G) in the *nad2* gene resulting in a non-synonymous substitution (Ser^13^>Gly^13^). Haplotype 1D3 differs a single nucleotide from haplotypes 1D1 and 1D2 in *nad4* on position 172 (C>T) which represents a synonymous substitution. Specimens with all three haplotypes were identified in greenhouse 2, whereas specimens from greenhouses 1, 4, 5 and 6 represented haplotypes 1D2 and 1D3. Only a single specimen could be collected in greenhouse 3 which represented the 1D2 haplotype (S5 Fig). Also found in haplogroup 1D2 are two specimens from Gloucester County, New Jersey, USA. Three specimens from this location were included in the study. The third specimen is a singleton and represents a unique haplotype in subgroup 1A, namely 1A3.

## Discussion

We generated complete mitogenome sequences of four *Anthonomus* species, and determined protein coding sequences of thirteen mitochondrial genes typically found in insects for 137 *Anthonomus* specimens from individual whole genome shotgun datasets. With these sequences, the phylogenetic relationship between these specimens was determined, and a haplotype analysis was performed to infer intra-species variation to identify possible linkages between *A*. *eugenii* specimens collected in the 2012 Dutch outbreak and specimens from the presumed center of origin and other outbreak locations in Italy and USA.

Combining *de novo* assembly of Illumina NextSeq data with a blastn approach allowed identification of putative mitochondrial contigs for individual insect specimens. These putative mitochondrial sequences could be used to create reliable reference mitochondrial genomes for *A*. *eugenii*, *A*. *pomorum*, *A*. *rubi*, and *A*. *rectirostris*. For *A*. *piri* however, a complete mitogenome could not be reconstructed as only limited amounts of sequence data were obtained for all three *A*. *piri* specimens. The majority of the *de novo* assembled sequences for these specimens were of human or bacterial origin. In *A*. *piri* specimen mtDNA157, two putative mitogenomic contigs were identified encoding *rnl* and *nad1*, and *atp6*, *cox3*, and *nad3* respectively. The orientation and organization of mitochondrial genes for these two contigs was the same as found in the other *Anthonomus* species, but as no complete mitogenome could be constructed, this species was excluded from further analysis.

The organization of mitochondrial elements (i.e. protein coding genes, tRNAs and rRNAs) in Coleopteran species is highly conserved, and the four sequenced *Anthonomus* species are no exception. The *trnI*-*trnQ* intergenic region shows the highest diversity between the species analyzed. For *A*. *pomorum*, *A*. *rubi*, and *A*. *rectirostris*, several repetitive sequences were identified in this region. Even though such repeat sequences were not obtained from the reference guided GRAbB assembly for the *A*. *eugenii* mitogenome, results from read-mapping and PCR verification suggest that an unresolved repeat sequence is present. This unresolved repeat sequence does not affect the analyses performed in this study as mitochondrial protein coding genes were used for the phylogenetic reconstruction and creation of the *A*. *eugenii* haplotype network.

### Phylogenetic reconstruction

The 13-gene phylogeny provides strong support for the species specific clades and shows monophyly for all species analyzed. Within *A*. *eugenii*, three specimens showed relative high diversity compared to the other 124 specimens. These specimens cluster as a sister group to the other *A*. *eugenii* specimens with 100% bootstrap support. In the haplotype analysis these specimens were assigned to haplogroup 3. Morphological characters of these specimens confirm their identify as *A*. *eugenii*. Our results further support the application of complete mitochondrial genomes to infer phylogenetic relations between insect specimens as was previously shown for stoneflies [35] and bush crickets [36].

### Haplotype network

A read-mapping approach was used to obtain the mitochondrial sequences of 135 *A*. *eugenii* specimens with >10x coverage, and a consensus mitogenome was obtained for the majority of specimens. However, eight *A*. *eugenii* specimens did not produce sufficient reads for reliable mapping to the reference mitogenome and these were excluded from the analysis. The diagnostic resolution of the thirteen mitochondrial protein coding gene sequences to infer population-level variation was far superior compared to the 658 bp standard *cox1* barcode sequence. With the barcoding fragment, only some major haplogroups can be differentiated (S6 Fig).

Mexican specimens represent the highest diversity of the *A*. *eugenii* specimens tested. The high level of diversity fits with the hypothesis that Mexico could be the center of origin for the species. Almost equally diverse are the specimens intercepted and sampled in New Jersey, USA. We hypothesize that the high level of diversity found in those samples is the direct result of repeated import from locations where the pest is naturally present (such as Mexico). This in contrast to the relative low level of diversity found in specimens intercepted from the Dominican Republic. This low level of diversity in Dominican Republic specimens could be explained by a single introduction to that country.

In the specimens collected from the Dutch outbreak, three highly similar, but unique, haplotypes were found. The “Dutch haplotype” 1D2 was also found in two specimens from Gloucester County, New Jersey, USA. As the Netherlands do not have pepper trade with New Jersey, it is unlikely that the source of the Dutch outbreak lies in New Jersey. It is more likely that the specimens sampled in New Jersey and those sampled in the Netherlands originate from a shared source that was not included in this study.

From this variation within the specimens sampled during the Dutch outbreak (i.e. three haplotypes separated by a single polymorphism), it is likely that infested material was introduced from a single source with multiple specimens representing the observed intra-population variation. The alternative, i.e. the introduction of a single female (and male) from which the variation within the Dutch specimens emerged *de novo* is unlikely. This also suggests that the greenhouse in which the highest diversity was found (i.e. greenhouse 2) could be the location where the pest was introduced first. in this scenario, specimens of the outbreak population in greenhouse 2 migrated to the other greenhouses and consequently represented less diversity.

Shortly after the Dutch outbreak in 2012, an *A*. *eugenii* outbreak was reported from Italy [15]. The haplotype found in the Italian specimens was the same as those found in the largest haplogroup representing specimens from the Dominican Republic. This haplotype is not linked to any of the Dutch haplotypes, suggesting that the Dutch and Italian outbreaks were not linked.

### Outbreak scenarios

During the Dutch *A*. *eugenii* outbreak, six greenhouses were affected in a 2 km radius in July to October 2012. The possibilities of infestation as a result of natural spread were assessed. *A*. *eugenii* has limited means of natural spread, and although the minimum temperature for flight is unknown, it is most likely above 15.0°C [37]. Flight activity increases with temperature. At temperatures of 25.0 to 28.8°C, half of the adults had initiated a flight and 90% at temperatures of 25.7 to 35.0°C [38]. Climatological data from the Hoek van Holland weather station (at ~5 km distance from the infested greenhouses) shows temperatures ranging from a nighttime low of 1.6 to a daytime high of 34.1°C with average nighttime and daytime temperatures of 12.5°C ±3.4 and 19.1°C ±4.5 respectively, during the period of infestation (S4 Table). Observations in 2013 and 2014 from New Jersey suggest that pepper weevils can fly as far as 1.5 miles and may be aided in their dispersal by prevailing winds and storm fronts [8]. Greenhouses are ventilated at the rooftop during the growing season and it is possible that adults have moved out of the infested greenhouse and into a nearby greenhouse.

Survival outdoors in northern Europe, however, is unlikely, given the fact that during a normal winter there are always periods of frost of -5.0°C or less. In Italy, survival during mild winters (with temperatures around or just below zero) is possible, whereas populations will die out when temperatures reach -5.0°C or less [19]. Overwintering of specimens inside greenhouses could be possible: during removal of the crops in fall, greenhouses are cleaned, but not meticulously and not all at the same time. It is unlikely, however, that an infestation will have persisted over more than a few growing seasons, without being noticed.

Most likely the incursions have taken place by transfer of *Anthonumus eugenii* adults originating from import produce. In the Netherlands *Anthonomus eugenii* has been intercepted as adults multiple times from vegetables (chili peppers, egg-plant fruits) imported from outside the EU (e.g. [39]), and it is common practice that pepper growers sort their own local produce as well as produce from others at their packaging station, directly in the vicinity of their crop. In Italy outbreaks in Lazio were allocated nearby the city of Fondi, a major distribution center for international vegetable produce [19].

## Conclusions

We successfully assembled and annotated the *A*. *eugenii* mitogenome, which was used to analyze an outbreak population. From this analysis we conclude that the outbreak, that affected six greenhouses in a 2 km radius, was the result of a single introduction with multiple specimens, likely via the import of infected produce. Other greenhouses were likely infested from the originally infested greenhouse by natural spread of the pest. The origin of the outbreak population could not be identified, but specimens with an identical haplotype were found in New Jersey, USA. It is unlikely that this locality represents the source of the Dutch outbreak. Mitochondrial haplotypes of specimens from an 2013 Italian outbreak location are different from those from the 2012 Dutch outbreak. Based on the similarities of the mitochondrial genomes of Italian specimens and specimens from the Dominican Republic, the latter country could represent the source of the Italian infestation. The mitochondrial genome is a useful tool to trace outbreak populations, but it relies on availability of specimens from the center of origin. To further improve the power of this track-an-trace tool, additional specimens from Mexico, Hawaii, and Southern USA states could be sequenced and included in the analysis. The methodology presented in this paper could prove valuable for other invasive pest species, such as the African fruit moth *Thaumatotibia leucotreta* and emerald ash borer *Agrilus planipennis*.

## Supporting information

S1 Fig Verification of circularity of the *A*. *eugenii* mitogenome. **Left** Eight primer pairs were designed to verify the circular conformation of the *A*. *eugenii* mitogenome. The corresponding amplicons, and their expected length based on the *in-silico* design, are shown on the *A*. *eugenii* specimen mtDNA140 mitogenome (MK654676). **Right** Gel image of amplicons obtained for the eight *A*. *eugenii* mitogenome primer pairs for specimen mtDNA140. All primer pairs resulted in amplicons corresponding to the expected amplicon length, except primer pair F6/R6. Where the amplicon length with this primer pair was expected to be 1,311 bp, a ~3.5 kbp fragment was obtained (arrow). It is hypothesized this is the result of a repeat sequence in the *trnI*–*trnQ* intergenic region which was not resolved in the *de novo* assembly.
(TIF)

S2 Fig Read coverage to the *A*. *eugenii* mitogenome reveals an unresolved repeat. The mitogenomic sequence MK654676 and associated annotations (genes, coding sequences, rRNAs and tRNAs) are shown as separate tracks. In addition, verification amplicons 1 to 8 are shown together with the mapping of NextSeq reads generated from *A*. *eugenii* specimen mtDNA140. A sharp increase in the read coverage in the *trnI*-*trnQ* intergenic region (amplicon 6) was observed (2,176x mean coverage compared to an overall mean of 1,201x). This strengthens the hypothesis that a repeat sequence is present in the *trnI*—*trnQ* intergenic region which was not resolved in the *de novo* assembly.
(TIF)

S3 Fig Haplotype network colored by specimens originating from Mexico. Median Joining haplotype network representing 127 *A*. *eugenii* specimens based on sequences of mitochondrial protein coding genes. Nodes in the network are colored based on the localities (states) where the specimens from Mexico were found. Black nodes represent hypothetical ancestors. Haplogroups are subdivided when they represent multiple localities of the same country. Marks on the branches indicate the number of mutations.
(TIF)

S4 Fig Haplotype network colored by specimens collected in USA. Median Joining haplotype network representing 127 *A*. *eugenii* specimens based on sequences of mitochondrial protein coding genes. Nodes in the network are colored based on the localities where the specimens from New Jersey (NJ) and Georgia (GA) were found. Black nodes represent hypothetical ancestors. Haplogroups are subdivided when they represent multiple localities of the same country. Marks on the branches indicate the number of mutations.
(TIF)

S5 Fig Detail of clustering of Dutch outbreak specimens in haplogroup 1. Nodes in the network are colored based on the Dutch localities of the samples following the 2012–2013 outbreak in the province of Zuid-Holland (ZH). Additionally, three specimens found in New Jersey (NJ) location 2 in Gloucester county are colored grey, as two of them share the main haplotype found in the Dutch outbreak population (1D2), while the third has haplotype 1A3. Black nodes represent hypothetical ancestors. Marks on the branches indicate the number of mutations.
(TIF)

S6 Fig Haplotype network of *A*. *eugenii* specimens based on DNA barcodes. Barcode sequences were defined as partial cox1 sequences flanked by PCR amplification primers LCO1490 and HCO2198 as described in EPPO standard PM7/129(1). These sequences were extracted in silico from the mitogenomic sequences of the 127 *A*. *eugenii* specimens included in this study. Nodes in the network are colored based on the origin of the samples, and black nodes represent hypothetical ancestors. Main groups as obtained with the thirteen mitochondrial protein coding sequences are shown.
(TIF)

S1 Table Specimens included in this study and associated metadata. Metadata per specimen include but are not limited to, species identity, collection source, country of origin, collection date, NextSeq yield, read coverage to reference mitogenomes and NCBI accessions for each of the protein coding genes assembled.
(XLSX)

S2 Table Primer sequences and thermocycler profiles used for the verification of circularity of the *A*. *eugenii* mitogenome. Primer sequences, corresponding thermocycler profiles and expected amplicon sizes are listed.
(XLSX)

S3 Table Haplotype information of *A*. *eugenii* specimens included in this study. The main haplogroup, subgroup, and haplotype are listed per *A*. *eugenii* specimen.
(XLSX)

S4 Table Climatological data from the Hoek van Holland weather station from 1 July—31 October 2012. The following abbreviations are used: YYYYMMDD = Date (YYYY = year MM = month DD = day); FG = Daily mean windspeed (in 0.1 m/s); TG = Daily mean temperature in (0.1 degrees Celsius); TN = Minimum temperature (in 0.1 degrees Celsius); TX = Maximum temperature (in 0.1 degrees Celsius); DR = Precipitation duration (in 0.1 hour); and RH = Daily precipitation amount (in 0.1 mm) (-1 for <0.05 mm).
(XLSX)

## References

[1] Elmore J.C., Davis A.C., and Campbell R.E., The pepper weevil. Technical bulletin United States Department of Agriculture, 1934(447).

[2] Riley D.G. and King E.G., Biology and management of pepper weevil Anthonomus eugenii Cano (Coleoptera: Curculionidae): a review. Trends Agri. Sci., 1994(2): p. 109–121.

[3] Walker C.M., The pepper weevil (Anthonomus aeneotinctus Champ.). USDA. Bur. Entomol. Bull., 1905(54): p. 43–48.

[4] Campbell R.E., Injuries to Peppers in California by Anthonomus Eugenii Cano. Journal of Economic Entomology, 1924 17(6): p. 645–647.

[5] Patrock R.J. and Schuster D.J., Feeding, oviposition and development of the pepper weevil, (Anthonomus eugenii Cano), on selected species of Solanaceae. Tropical Pest Management, 1992 38(1): p. 65–69.

[6] OEPP/EPPO. EPPO Global Database. 2019 [cited 2019 12 April]; Available from: https://gd.eppo.int/.

[7] Goff C.C. and Wilson J.W., The pepper weevil. Florida Agricultural Experiment Station Bulletin, 1937(310): p. 1–12.

[8] Ingerson-Mahar J., Eichinger B., and Holmstrom K., How Does Pepper Weevil (Coleoptera: Curculionidae) Become an Important Pepper Pest in New Jersey? Journal of Integrated Pest Management, 2015 6(1): p. 23–23.

[9] Hammes C. and Putoa R., *Catalogue des insectes et acariens d'intérêt agricole de Polynésie française*. 1986, ORSTOM: Papeete p. 260 p. multigr.

[10] Abreu E. and Cruz C., The occurrence of the pepper weevil, Anthonomus eugenii in Puerto Rico J. Agric. Univ. P. R., 1985 69: p. 223–224.

[11] Serra C.A., et al, *Impacts of recently emerged invasive exotic species and major threats to the Dominican agriculture*. 2011.

[12] Costello R.A. and Gillespie D.R., The Pepper weevil, Anthonomus eugenii Cano as greenhouse pest in Canada. IOBC PWRS Bulletin, 1993 16(2): p. 31–34.

[13] CFIA, RMD-10-28: Anthonomus eugenii (pepper weevil). Pest Risk Management Document, 2011.

[14] NPPO-NL, First report of Anthonomus eugenii in the Netherlands. EPPO Reporting Service, 2012. 10(2012/203).

[15] Speranza S., et al, First Record of Anthonomus eugenii (Coleoptera: Curculionidae) in Italy. Florida Entomologist, 2014 97(2): p. 844–845.

[16] van der Gaag D. and Loomans A.J.M., Pest Risk Analysis for Anthonomus eugenii v.3. Netherlands Food and Consumer Product Safety Authority, Utrecht, 2013: p. 64 p.

[17] De Goffau L.J.W., Larvae of Anthonomus cf. eugenii in fruits of eggplants from the Dominican Republic. Verslagen en Mededelingen Plantenziektenkundige Dienst Wageningen, 2000(201): p. 56.

[18] NPPO-NL, Anthonomus eugenii eradicated from the Netherlands. EPPO Reporting Service, 2014. 2(2014/024).

[19] Anonymous. Relazione sull'infestatatione da Anthonomus eugenii (Cano) nel territorio Laziale (2013–2018). 2018; Available from: http://www.agricoltura.regione.lazio.it/binary/prtl_sfr/tbl_misure/ANTHEU_REL_2013_2018.pdf.

[20] Fenn J.D., et al, A preliminary mitochondrial genome phylogeny of Orthoptera (Insecta) and approaches to maximizing phylogenetic signal found within mitochondrial genome data. Mol Phylogenet Evol, 2008 49(1): p. 59–68. 10.1016/j.ympev.2008.07.004 18672078

[21] Hua J., et al, Phylogenetic analysis of the true water bugs (Insecta: Hemiptera: Heteroptera: Nepomorpha): evidence from mitochondrial genomes. BMC evolutionary biology, 2009 9: p. 134–134. 10.1186/1471-2148-9-134 19523246 PMC2711072

[22] Blacket M.J., et al, Screening mitochondrial DNA sequence variation as an alternative method for tracking established and outbreak populations of Queensland fruit fly at the species southern range limit. Ecol Evol, 2017 7(8): p. 2604–2616. 10.1002/ece3.2783 28428851 PMC5395428

[23] Battaglia V., et al, The Worldwide Spread of the Tiger Mosquito as Revealed by Mitogenome Haplogroup Diversity. Frontiers in genetics, 2016 7: p. 208–208. 10.3389/fgene.2016.00208 27933090 PMC5120106

[24] Friedman J.R. and Nunnari J., Mitochondrial form and function. Nature, 2014 505(7483): p. 335–43. 10.1038/nature12985 24429632 PMC4075653

[25] Taanman J.W., The mitochondrial genome: structure, transcription, translation and replication. Biochim Biophys Acta, 1999 1410(2): p. 103–23. 10.1016/s0005-2728(98)00161-3 10076021

[26] Smith D.R., The past, present and future of mitochondrial genomics: have we sequenced enough mtDNAs? Briefings in Functional Genomics, 2016 15(1): p. 47–54. 10.1093/bfgp/elv027 26117139 PMC4812591

[27] Gillett C.P.D.T., et al, Bulk De Novo Mitogenome Assembly from Pooled Total DNA Elucidates the Phylogeny of Weevils (Coleoptera: Curculionoidea). Molecular Biology and Evolution, 2014 31(8): p. 2223–2237. 10.1093/molbev/msu154 24803639 PMC4104315

[28] Clark W.E. and Burke H.R., The species of Anthonomus germar (Coleoptera: Curculionidae) associated with plants in the family Solanaceae. Southwestern Entomol. Suppl., 1996 19: p. 1–114.

[29] Folmer O., et al, DNA primers for amplification of mitochondrial cytochrome c oxidase subunit I from diverse metazoan invertebrates. Mol Mar Biol Biotechnol, 1994 3(5): p. 294–9. 7881515

[30] EOPP/EPPO, PM 7/129 (1) DNA barcoding as an identification tool for a number of regulated pests. EPPO Bulletin, 2016 46(3): p. 501–537.

[31] Katoh K. and Standley D.M., MAFFT multiple sequence alignment software version 7: improvements in performance and usability. Molecular biology and evolution, 2013 30(4): p. 772–780. 10.1093/molbev/mst010 23329690 PMC3603318

[32] Brankovics B., et al, GRAbB: Selective Assembly of Genomic Regions, a New Niche for Genomic Research. PLoS computational biology, 2016 12(6): p. e1004753–e1004753. 10.1371/journal.pcbi.1004753 27308864 PMC4911045

[33] Bernt M., et al, MITOS: improved de novo metazoan mitochondrial genome annotation. Mol Phylogenet Evol, 2013 69(2): p. 313–9. 10.1016/j.ympev.2012.08.023 22982435

[34] Leigh J.W. and Bryant D., popart: full-feature software for haplotype network construction. Methods in Ecology and Evolution, 2015 6(9): p. 1110–1116.

[35] Wang Y., Cao J.-J., and Li W.-H., Complete Mitochondrial Genome of Suwallia teleckojensis (Plecoptera: Chloroperlidae) and Implications for the Higher Phylogeny of Stoneflies. International journal of molecular sciences, 2018 19(3): p. 680. 10.3390/ijms19030680 PMC5877541 29495588

[36] Shaoli M., et al, The complete mitochondrial genome of Xizicus (Haploxizicus) maculatus revealed by Next-Generation Sequencing and phylogenetic implication (Orthoptera, Meconematinae). ZooKeys, 2018(773): p. 57–67. 10.3897/zookeys.773.24156 30026660 PMC6048180

[37] Smith I.M., et al, *Quarantine Pests for Europe–Data Sheets on quarantine pests for the European Union and for the European and Mediterranean Plant Protection Organization*. 2nd ed 1996: CABI, Wallingford, United Kingdom.

[38] Jones D. and Sterling W.L., Rate and Thresholds of Boll Weevil 1 Locomotory Activity in Response to Temperature 2. Environmental Entomology, 1979 8(5): p. 874–878.

[39] EU. Interceptions of harmful organisms in imported plants and other objects. Available from: https://ec.europa.eu/food/plant/plant_health_biosecurity/europhyt/interceptions_en.

