# Supplementary figures and images for: Tracking outbreak populations of the pepper weevil Anthonomus eugenii (Coleoptera; Curculionidae) using complete mitochondrial genomes
Source: PLoS One. 2019 Aug 14;14(8):e0221182. doi: 10.1371/journal.pone.0221182 (PMC6693850; doi:10.1371/journal.pone.0221182)

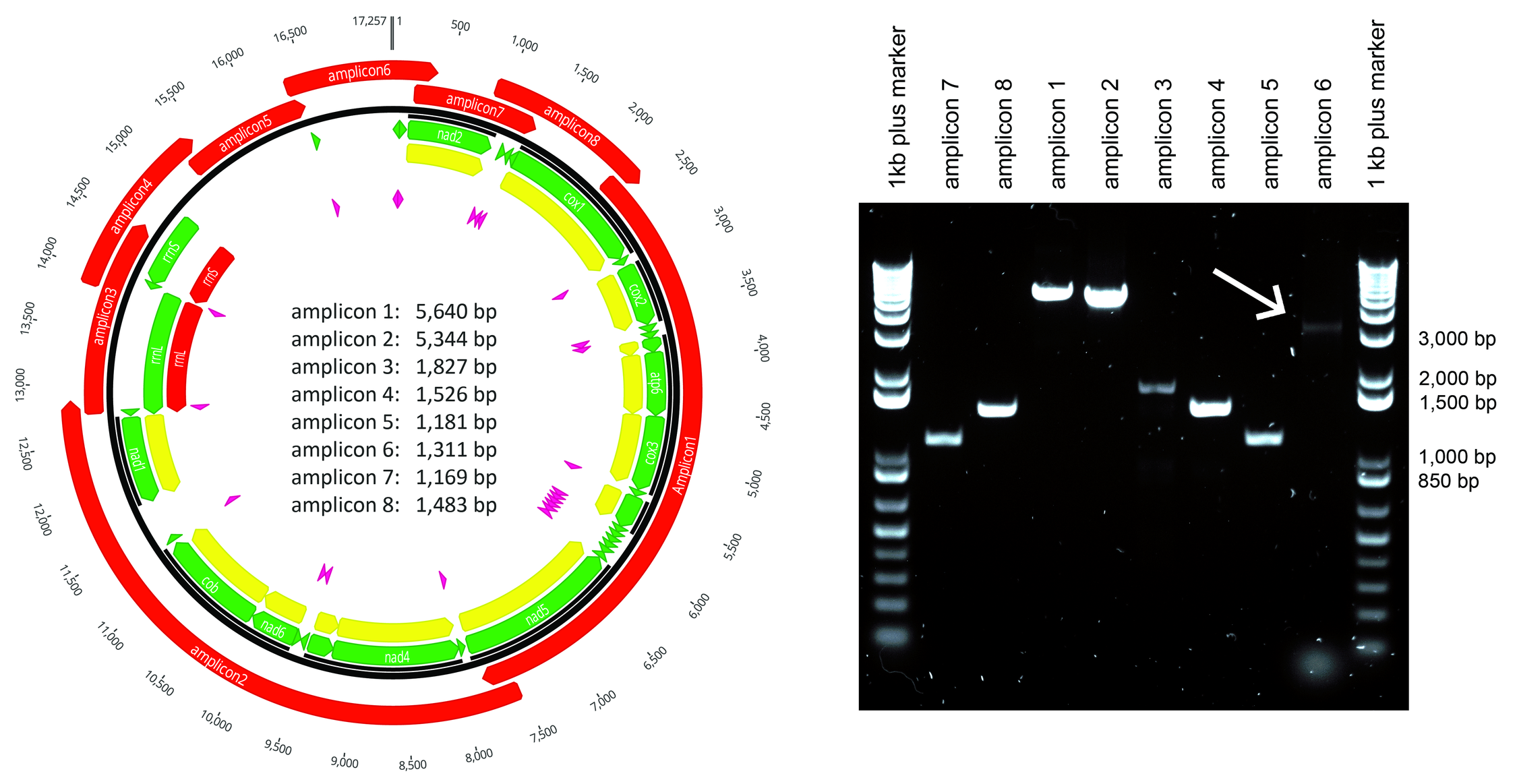

Supplement: S1 Fig — Left Eight primer pairs were designed to verify the circular conformation of the A. eugenii mitogenome. The corresponding amplicons, and their expected length based on the in-silico design, are shown on the A. eugenii specimen mtDNA140 mitogenome (MK654676). Right Gel image of amplicons obtained for the eight A. eugenii mitogenome primer pairs for specimen mtDNA140. All primer pairs resulted in amplicons corresponding to the expected amplicon length, except primer pair F6/R6. Where the amplicon length with this primer pair was expected to be 1,311 bp, a ~3.5 kbp fragment was obtained (arrow). It is hypothesized this is the result of a repeat sequence in the trnI–trnQ intergenic region which was not resolved in the de novo assembly. (TIF) [file pone.0221182.s001.tif]

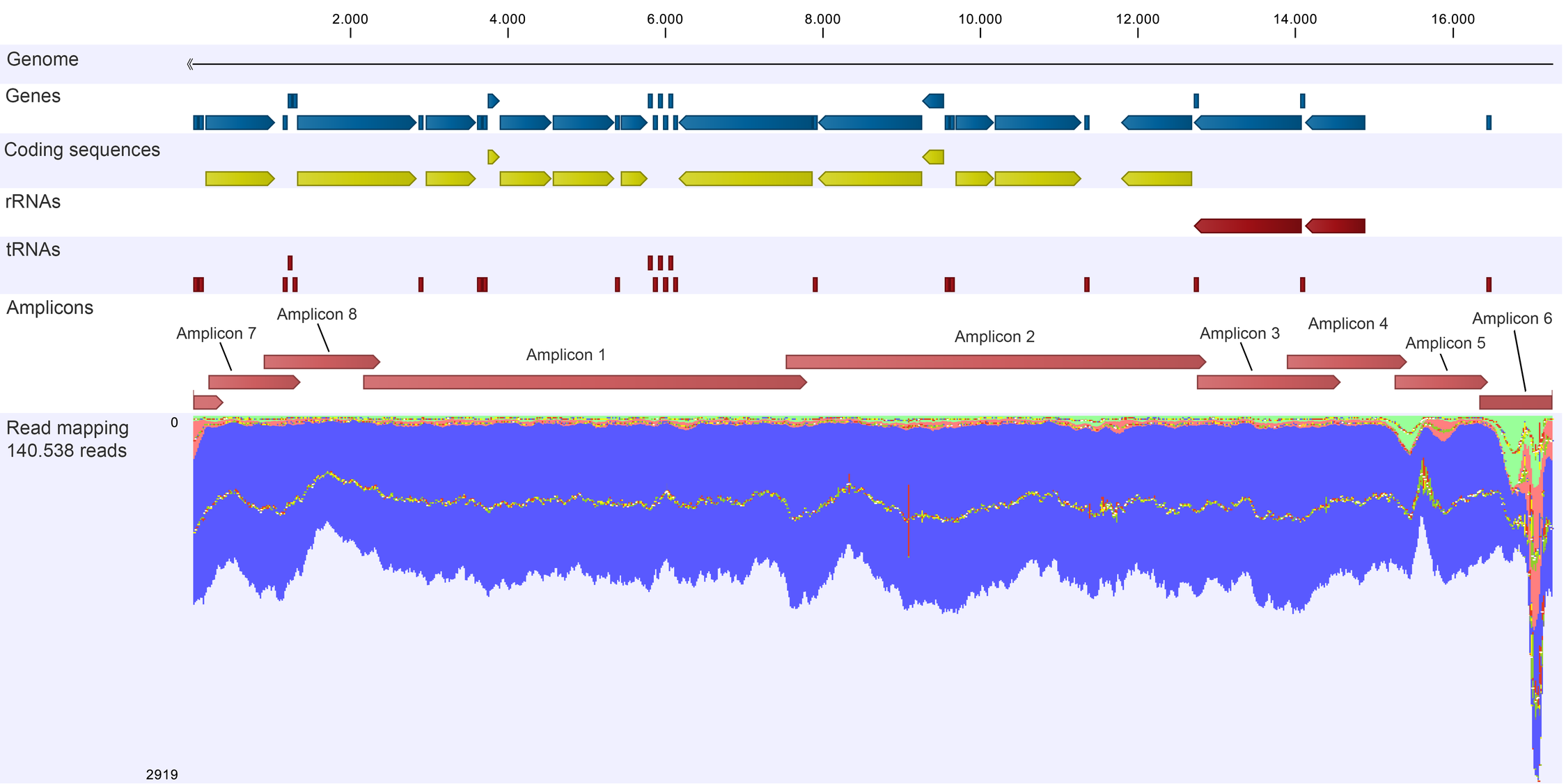

Supplement: S2 Fig — The mitogenomic sequence MK654676 and associated annotations (genes, coding sequences, rRNAs and tRNAs) are shown as separate tracks. In addition, verification amplicons 1 to 8 are shown together with the mapping of NextSeq reads generated from A. eugenii specimen mtDNA140. A sharp increase in the read coverage in the trnI-trnQ intergenic region (amplicon 6) was observed (2,176x mean coverage compared to an overall mean of 1,201x). This strengthens the hypothesis that a repeat sequence is present in the trnI—trnQ intergenic region which was not resolved in the de novo assembly. (TIF) [file pone.0221182.s002.tif]

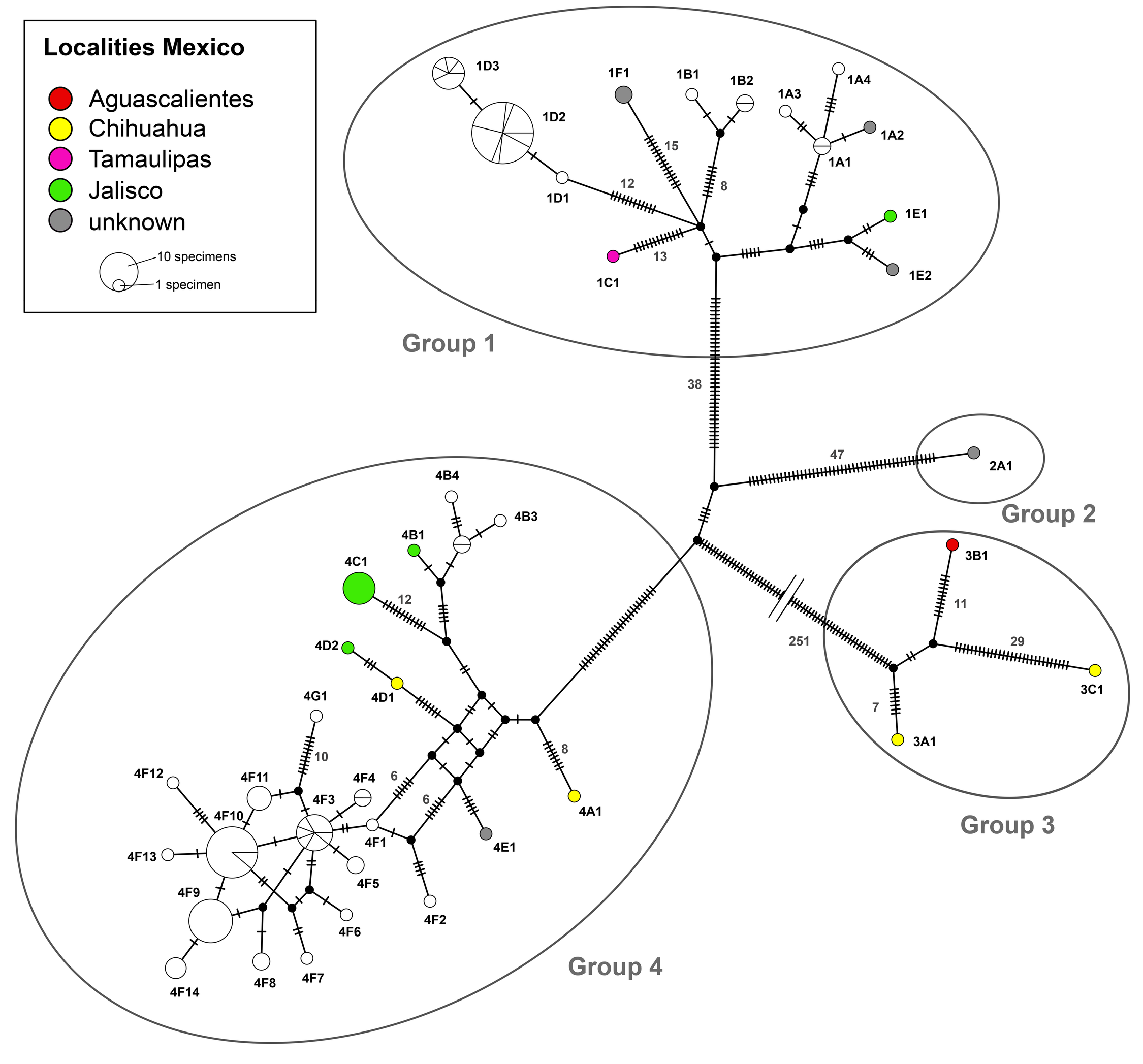

Supplement: S3 Fig — Median Joining haplotype network representing 127 A. eugenii specimens based on sequences of mitochondrial protein coding genes. Nodes in the network are colored based on the localities (states) where the specimens from Mexico were found. Black nodes represent hypothetical ancestors. Haplogroups are subdivided when they represent multiple localities of the same country. Marks on the branches indicate the number of mutations. (TIF) [file pone.0221182.s003.tif]

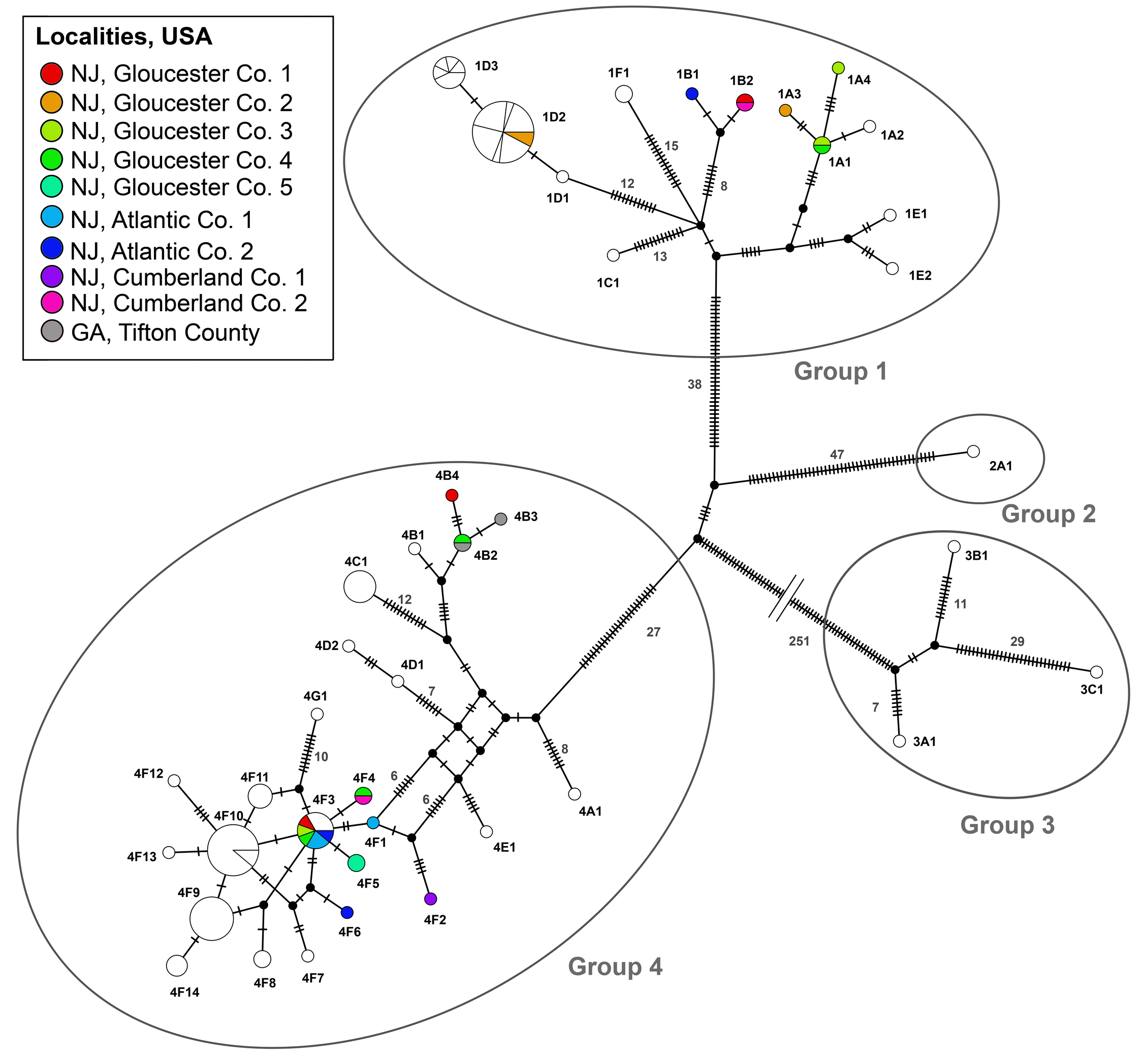

Supplement: S4 Fig — Median Joining haplotype network representing 127 A. eugenii specimens based on sequences of mitochondrial protein coding genes. Nodes in the network are colored based on the localities where the specimens from New Jersey (NJ) and Georgia (GA) were found. Black nodes represent hypothetical ancestors. Haplogroups are subdivided when they represent multiple localities of the same country. Marks on the branches indicate the number of mutations. (TIF) [file pone.0221182.s004.tif]

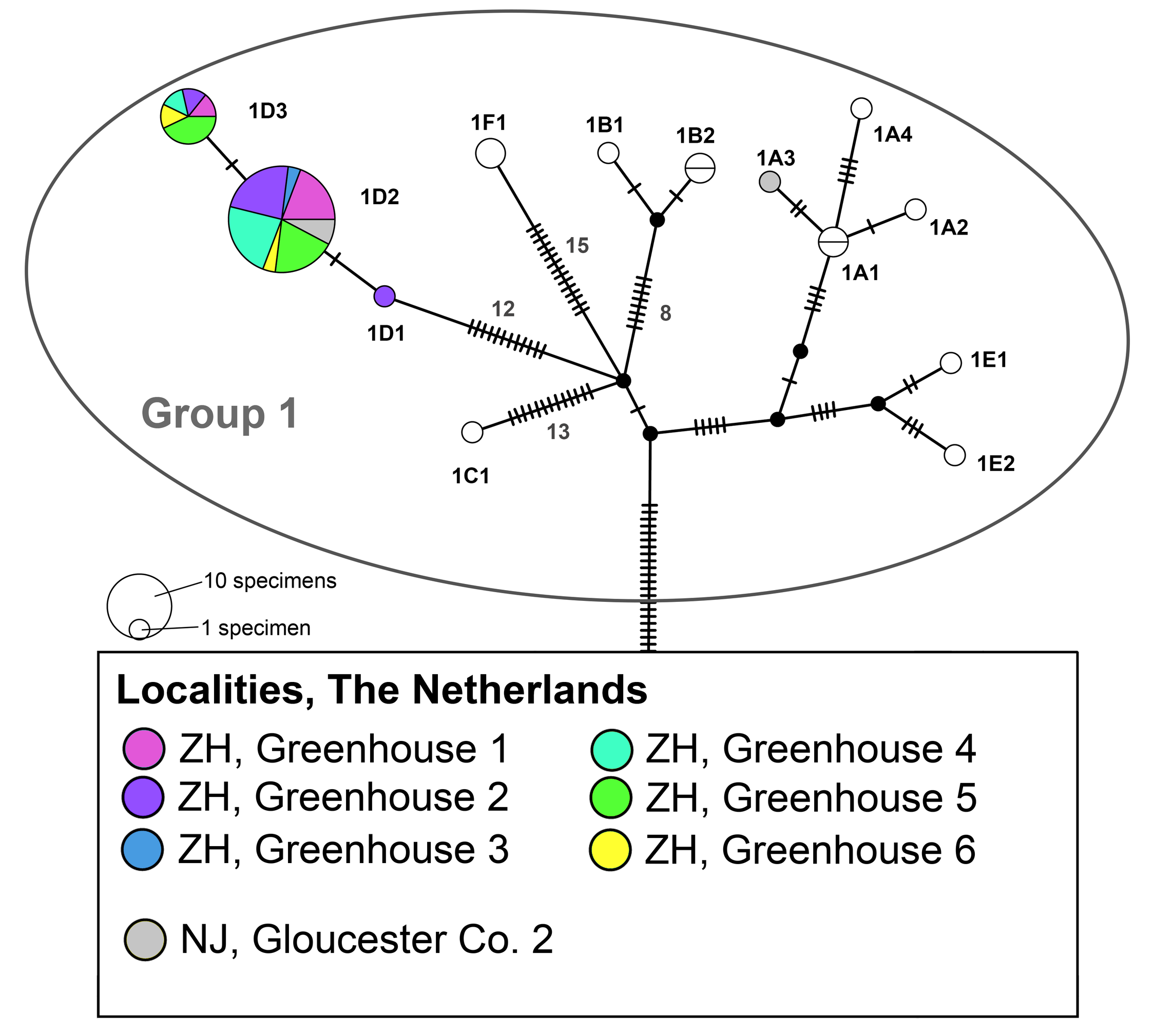

Supplement: S5 Fig — Nodes in the network are colored based on the Dutch localities of the samples following the 2012–2013 outbreak in the province of Zuid-Holland (ZH). Additionally, three specimens found in New Jersey (NJ) location 2 in Gloucester county are colored grey, as two of them share the main haplotype found in the Dutch outbreak population (1D2), while the third has haplotype 1A3. Black nodes represent hypothetical ancestors. Marks on the branches indicate the number of mutations. (TIF) [file pone.0221182.s005.tif]

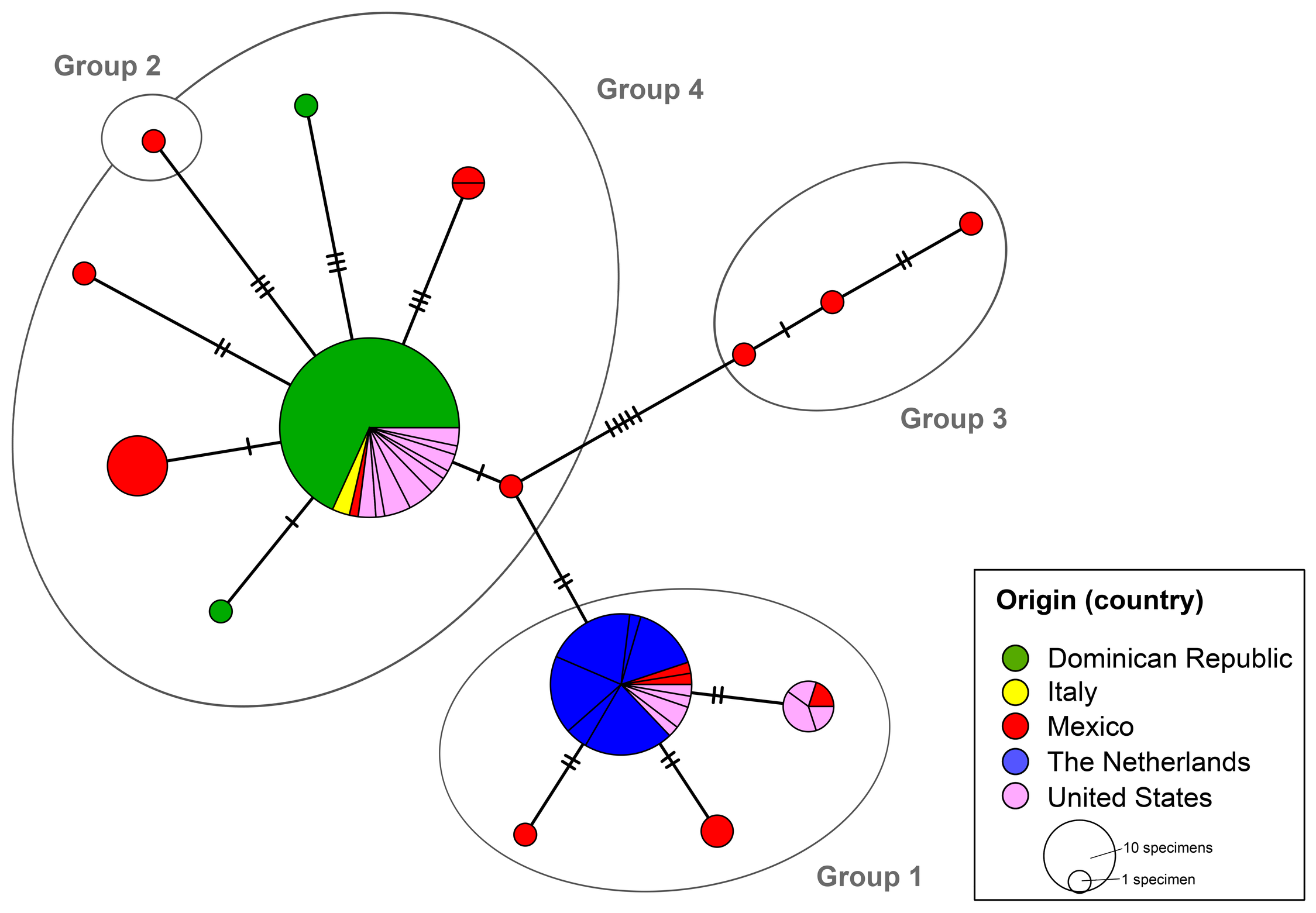

Supplement: S6 Fig — Barcode sequences were defined as partial cox1 sequences flanked by PCR amplification primers LCO1490 and HCO2198 as described in EPPO standard PM7/129(1). These sequences were extracted in silico from the mitogenomic sequences of the 127 A. eugenii specimens included in this study. Nodes in the network are colored based on the origin of the samples, and black nodes represent hypothetical ancestors. Main groups as obtained with the thirteen mitochondrial protein coding sequences are shown. (TIF) [file pone.0221182.s006.tif]
